# Left atrial fat with cardiac MR

**DOI:** 10.1186/1532-429X-11-S1-P128

**Published:** 2009-01-28

**Authors:** Dana C Peters, James W Goldfarb, Reza Nezafat, Nisha I Parikh, Michael L Chuang, Warren J Manning

**Affiliations:** 1 Beth Israel Deaconess Medical Center and Harvard Medical School, Boston, MA USA; 2 Department of Research and Education,, Saint Francis Hospital, Roslyn, NY USA

**Keywords:** Atrial Fibrillation, Left Atrium, Atrial Fibrillation Patient, Healthy Adult Subject, Ganglionated Plexi

## Introduction

It has been proposed that the ganglionated plexi (GP) residing in the fat pads surrounding the left atrium (LA) may be involved in the genesis of atrial fibrillation (AF) [1]. Appropriate ablation of these targets may be a treatment of AF [2]. The gross anatomy of the GPs embedded in peri-atrial fat has been described and classified into five regions [3]: the superior right atrial GP, the superior left atrial GP, the posterior right atrial GP, the posteromedial left atrial GP, and posterolateral left atrial GP. Cardiac MR (CMR) is unique among non-invasive imaging modalities, providing excellent depiction of fat, and can contribute by identifying these fat pads.

## Methods

**Figure 1:**
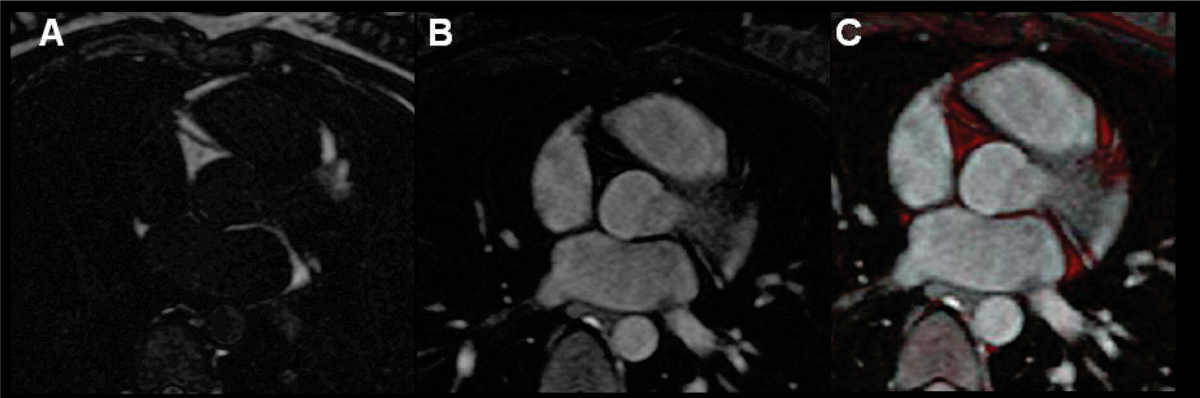
**A) Fat image; B) blood pool image; C) fusion of fat and blood pool with fat highlighted in red**.

Six healthy adult subjects (age = 24 +/- 4, 3 M) underwent CMR with two high spatial resolution 3D free-breathing sequences. Scan parameters in common for both sequences were: 3D gradient echo, axial, navigator-gated and ECG-gating to diastole, TR/TE/θ = 5.3/2.5/20°, 32 views per segment, 16 Nz, 1.3 × 1.3 × 5 mm spatial resolution. The first sequence used an inversion pulse with a TI of 400 ms, to null blood and myocardium, while fat is bright (Figure 1A). The second sequence used a T2-prep (20 ms), and fat-suppression to visualize the pulmonary veins (PVs) and the LA only (Figure 1B). No exogenous contrast was used. These two free-breathing sequences can be fused, with color or non-color highlighting, to demonstrate fat in the context of anatomy (Figure 1C).

## Results

**Figure 2:**
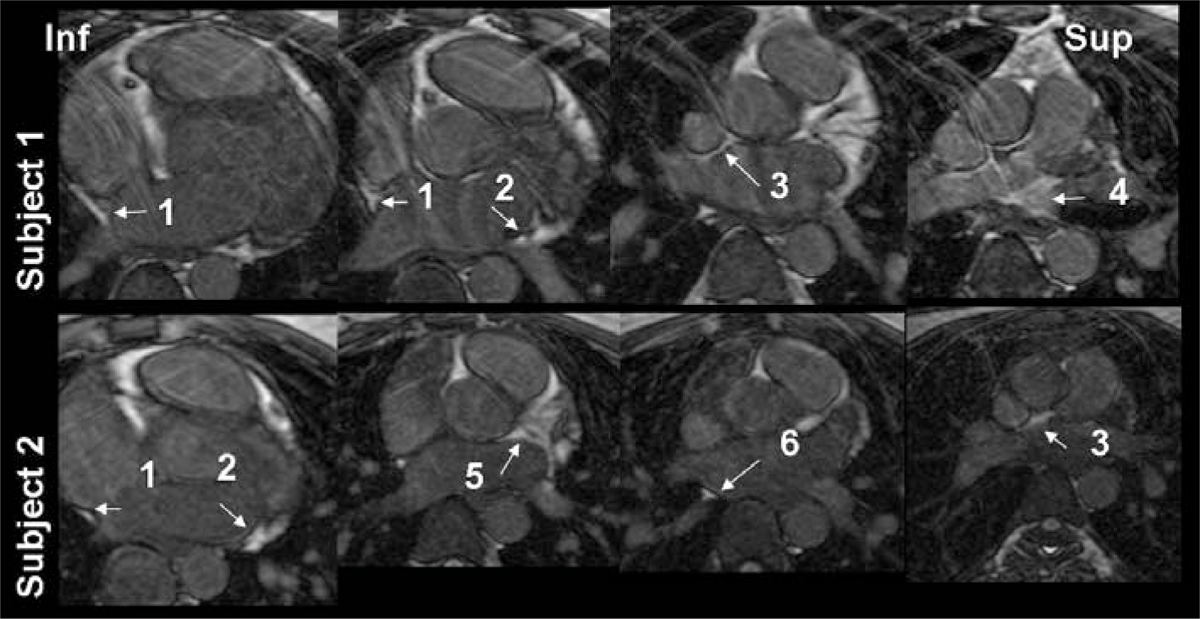
**Four axial slices (inferior to superior) of the LA are shown in two subjects, with fat and blood pool images combined, and fat highlighted as brighter signal**. Fat was present below and adjacent to the right inferior PV (labeled #1 in Figure 2, the posteromedial left atrial GP), below the left inferior PV (#2 the posterolateral left atrial GP), adjacent to the right superior PV near the superior vena cava (#3, the superior right atrial GP), above the left superior PV (#4, the superior left atrial GP), along the anterior wall (#5), and above the right inferior PV (#6, the posterior right atrial GP).

Figure 2 shows representative slices (from inferior to superior) from two healthy subjects, with arrows pointing to typical locations of fat. All subjects had fat in proximity to locations of the posterolateral left atrial GP, posterior right atrial GP, and superior right atrial GP, 4/6 subjects had fat around the superior left atrial GP, and 5/6 subjects had fat around the posteromedial left atrial GP.

## Discussion and conclusion

This preliminary CMR study shows the ubiquity of fat around the LA and adjacent to the PV ostia, concentrated in previously described locations. Other CMR methods for detecting fat – from simple 2D balanced SSFP slices to 3-point Dixon techniques may also be useful. The ability to detect regional fat distribution may contribute to planning for ablation in AF patients.
